# Injectable kaempferol-loaded fibrin glue regulates the metabolic balance and inhibits inflammation in intervertebral disc degeneration

**DOI:** 10.1038/s41598-023-47375-3

**Published:** 2023-11-15

**Authors:** Wenshuo Gao, Jianhang Bao, Yujun Zhang, Du He, Liangping Zhang, Jun Zhang, Hao Pan, Dong Wang

**Affiliations:** 1 Department of Orthopaedics, Hangzhou TCM Hospital Affiliated to Zhejiang Chinese Medical University (Hangzhou Hospital of Traditional Chinese Medicine), Hangzhou, 310000 Zhejiang People’s Republic of China; 2 Department of Orthopaedics, Hangzhou Dingqiao Hospital, Huanding Road NO 1630, Hangzhou, 310021 Zhejiang People’s Republic of China; 3 Institute of Orthopaedics and Traumatology, Hangzhou Traditional Chinese Medicine Hospital Affiliated to Zhejiang Chinese Medical University, Tiyuchang Road NO 453, Hangzhou, 310007 Zhejiang People’s Republic of China; 4 Department of General Surgery, Institute of Orthopaedics and Traumatology, Hangzhou Dingqiao Hospital, Huanding Road NO 1630, Hangzhou, 310021 Zhejiang People’s Republic of China; 5 Department of Rehabilitation Medicine, The First Affiliated Hospital of Hebei North University, Zhangjiakou, 075000 Hebei People’s Republic of China; 6 Department of Orthopaedics, Yiwu Central Hospital, Yiwu, 322000 Zhejiang People’s Republic of China

**Keywords:** Biological techniques, Medical research

## Abstract

To construct an injectable fibrin glue system loaded with kaempferol (FG@F) to improve the bioavailability of kaempferol and observe its efficacy in the treatment of intervertebral disc degeneration (IVDD). Kaempferol-loaded fibrin glue was first synthesized in advance. Subsequently, the materials were characterized by various experimental methods. Then, nucleus pulposus cells (NPCs) were stimulated with lipopolysaccharide (LPS) to establish a degenerative cell model, and the corresponding intervention treatment was conducted to observe the effect in vitro. Finally, the tail disc of rats was punctured to establish a model of IVDD, and the therapeutic effect of the material in vivo was observed after intervertebral disc injection. The FG@F system has good injectability, sustained release and biocompatibility. This treatment reduced the inflammatory response associated with IVDD and regulated matrix synthesis and degradation. Animal experimental results showed that the FG@F system can effectively improve needle puncture-induced IVDD in rats. The FG@F system has better efficacy than kaempferol or FG alone due to its slow release and mechanical properties. The drug delivery and biotherapy platform based on this functional system might also serve as an alternative therapy for IVDD.

## Introduction

Intervertebral disc degeneration (IVDD) is a chronic degenerative disease^1,2^. IVDD is primarily caused by catabolic and anabolic disorders as well as changes in the microenvironment^3^. Through protection of nucleus pulposus cells (NPCs) and restoration of the IVDD microenvironment, IVDD treatment effectiveness can be increased^4,5^. Pyroptosis is a newly discovered inflammatory programmed cell death mode that can be triggered by the NLRP3 inflammasome and accompanied by the release of a large number of proinflammatory factors^6^. This process contributes to the gradual loss of extracellular matrix (ECM), leading to senescence and death of NPCs^7^. In addition, during IVDD, the abnormal expression of ADAMTS-5 and collagen II degradation disrupt the ECM balance by reducing the secretion of type II collagen from NPCs^8,9^. A cascade of exacerbated reactions occurs as a result of the remodelling of the IVDD microenvironment, as well as the accumulation of inflammatory factors and the death of NPCs. To alleviate IVDD, researchers need to restore the healthy IVDD microenvironment and protect NPCs^6,10^.

Kaempferol is a flavonoid that is slightly soluble in water, is soluble in organic solvents such as hot ethanol and DMSO, and has suitable anti-inflammatory and antioxidant effects^11,12^. Studies have shown that kaempferol exerts its anti-inflammatory effects by inhibiting the TLR4/NF-ĸB signalling pathway^13^. However, the role of kaempferol in IVDD and its related mechanisms are still rarely studied. Moreover, this simple drug is easily degraded when injected into the body, and it is difficult to exert lasting drug effects^14,15^.

Drug delivery systems are widely used in the treatment of many diseases and have the advantages of specific targeting, high biocompatibility and controlled release^16^. Fibrin glue (FG), a drug carrier consisting of fibrinogen solution and thrombin solution, is used for haemostasis, wound healing, and bone regeneration^17^. Studies have shown that FG, when used as a scaffold, can improve cell survival, proliferation, differentiation, and matrix synthesis and is a promising drug delivery system^18^.

**Scheme 1:**
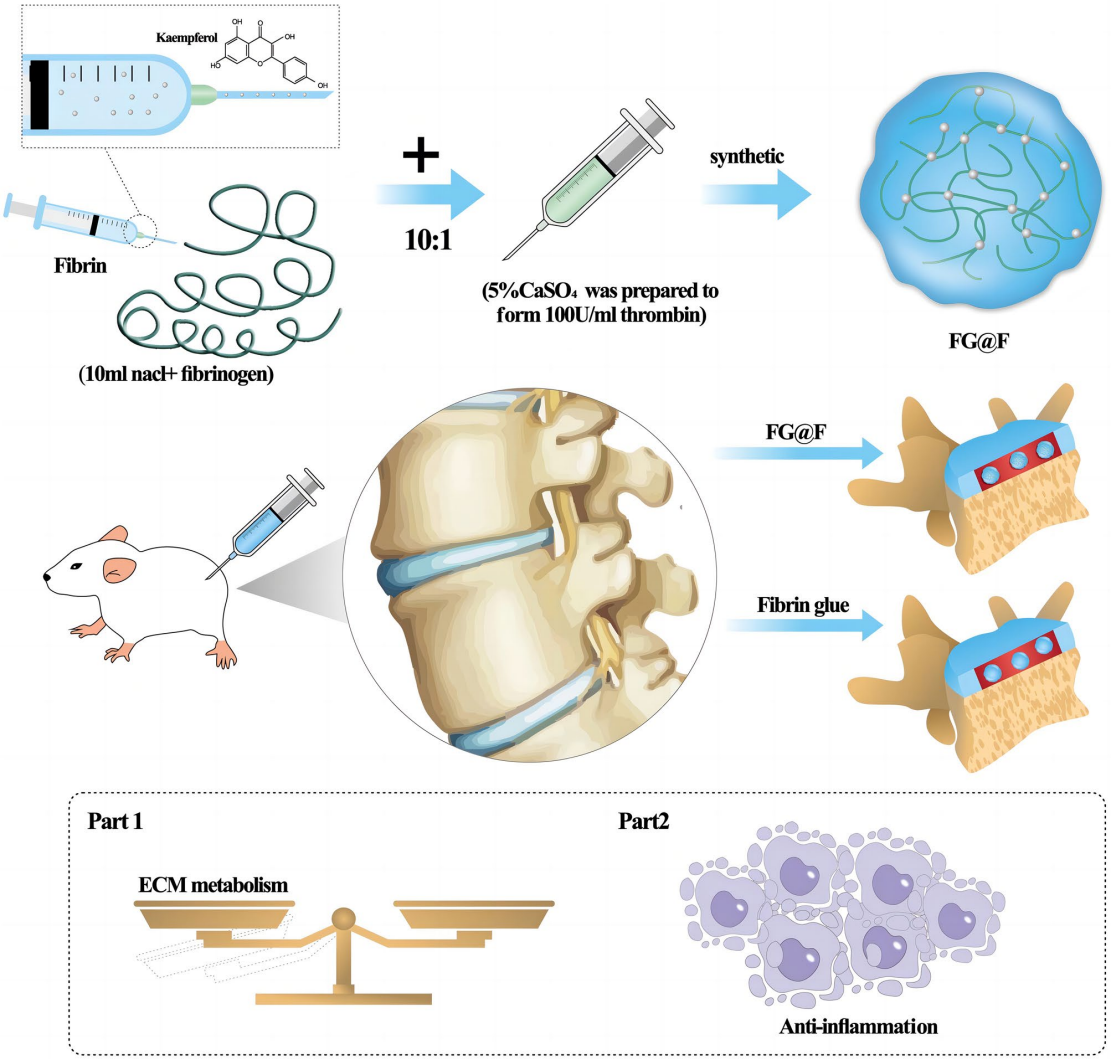
FG@F and FG synthesis and its mechanisms in the treatment of IVDD.

This study used resurrection lily phenol for optimal disc metabolic regulation. Fibrin glue was used to assist NPCs in combating inflammation by exerting optimal therapeutic properties and biological compatibility. By combining galanga resurrection lily phenol with fibrin glue, we constructed an injectable drug fibrin glue system to effectively restore the IVDD microenvironment and protect NPCs from inflammation after IVDD. Compared with traditional scaffolds, fibrin glue uses natural biological materials, does not contain toxic crosslinking agents, and has suitable anti-inflammatory and pro-proliferative effects^19–21^. FG@F was proven to be injectable and stable in vitro, maintaining a stable elastic solid morphology at 37 °C. The drug release curve showed that the fibrin glue could sustainably release kaempferol to exert a long-lasting drug effect. In addition, fibrin glue was examined for its effects on matrix catabolism and inflammation in degenerative NPCs. By using a rat tail disc degeneration model, we confirmed that fibrin glue could alleviate ECM degradation and promote ECM regeneration (Scheme 1). This study developed a novel IVDD bioglue to modulate the microenvironment, treat IVDD, and deliver kaempferol.

## Materials and methods

### Synthesis of FG@F

Fibrinogen was selected from bovine fibrinogen produced by Yeasen Biotechnology Co. (China). Fibrin was dissolved using 0.9% NaCl at 35 mg/10 ml. Thrombin was purchased from Zhejiang Hangkang Pharmaceutical Co., Ltd. (5000 U). A thrombin solution of 100 U/ml was prepared using 5% CaSO_4_. The fibrin glue was prepared with a 1:10 thrombin:fibrinogen ratio. The drug-loaded fibrin glue was prepared by mixing kaempferol (MedChemExpress, USA) with fibrinogen at a ratio of 1:10, and then, thrombin was injected into the mixed liquid for crosslinking.

### Physicochemical and multifunctional properties

#### Characterizations of FG@F

The porous morphology of FG@F was observed using scanning electron microscopy (SEM) (Hitachi SU8010, Japan). For determination of the rheological properties of the scaffolds (Gʹ and Gʹʹ), a rheometer (HAAKE MARS60, Germany) was employed. Using strain and frequency constants of 1% and 1 Hz, we investigated the effects of temperature on FG@F ranging from 4 to 50 °C. The injectability of biological scaffolds was determined by measuring viscosity changes with shear rate. All experiments were repeated three times.

#### Profile of kaempferol release from FG@F

Kaempferol release profiles were determined using Varioskan LUX (Thermo Scientific, USA). Briefly, 6 mg of kaempferol preparation was added to 3 ml of FG@F. The samples were immersed in 5 ml of PBS at 37 °C and replaced with 300 µl of fresh PBS at 12 h, 1 day, 2 days, 4 days, 8 days, and 12 days after the start of incubation. The sample absorbance was detected at 435 nm, and 6 mg kaempferol was added to 3 ml of PBS to determine the absorbance of the stock solution. Then, the absorbance value determined by the sample was compared with the absorbance of the stock solution to calculate the percentage of kaempferol release. All experiments were repeated three times.

#### Cytotoxicity and proliferation assays

NPCs were obtained from nucleus pulposus tissue isolated from tail discs of 4-week-old male Sprague–Dawley (SD) rats and purchased from Zhejiang Medical College (Hangzhou, China). Primary NPCs were obtained by continuous digestion with collagenase II (Solaibao, China) at 37 °C for 4 h, filtered through a sieve and centrifuged at 1000 rpm for 5 min. The isolated NPCs were cultured in Dulbecco’s modified Eagle’s medium (DMEM) with 10% FBS in a humidified incubator with 5% CO_2_. The medium was changed every other day. In the next experiment, we used cells from the second passage. The cytotoxicity and proliferation of FG and FG@F cultured for 1, 3, and 5 days were evaluated using a CCK-8 assay kit (Boster, Wuhan, China). Absorbance was measured at 450 nm after 10 µl of CCK-8 solution was added to the NPCs using Varioskan LUX (Thermo, USA). For determination of the distribution of living cells, a Calcein/PI Cell Viability/Cytotoxicity Assay Kit (Beyotime, China) was used. For fluorescence images, NPCs were incubated with calcein-AM (1 M) for 30 min in a 37 °C incubator. All experiments were repeated three times.

#### Immunofluorescence

The cells were washed three times with PBS and fixed for 15 min with precooled methanol at − 20 °C. After permeabilization of the cell membranes with 0.5% Triton X-100 for 20 min, the cells were washed again with PBS 3 times. We blocked the cells with 5% BSA for 30 min, added the appropriate primary antibody to the cells and incubated them at 4 °C overnight. The primary antibody information was as follows: rabbit anti-collagen II (1:500; Proteintech), rabbit anti-ADAMTS-5 (1:500, Proteintech), rabbit anti-NLRP3 (1:500, Proteintech) and rabbit anti-IL-1β (1:500, Proteintech). After three washes with PBS, we added goat anti-rabbit Alexa Fluor 488 and 594 antibodies (Beyotime, China). After 10 min, the samples were stained with DAPI (Beyotime, China) at room temperature. The above experiments were performed three times with a fluorescence microscope (Olympus BX51, Japan) and repeated three times.

#### Cellular treatments and real-time PCR (qPCR)

Cells were divided into five groups: control (Con group), 24 h LPS treatment + 1 h ATP treatment (LPS group), 24 h LPS treatment + 1 h ATP treatment + kaempferol (F group), 24 h LPS treatment + 1 h ATP treatment + FG (FG group) and 24 h LPS treatment + 1 h ATP treatment + FG@F (FG@F group). Cells in the F and FG groups were pretreated with kaempferol for 2 h before the addition of 2 µg/ml LPS (MedChemExpress, USA) and 5 mM ATP (MedChemExpress, USA). We extracted RNA from lysed NPCs with TRIzol reagent (Invitrogen, USA) and an RNA Purification Kit (CW0581, Kangwei) and reverse transcribed the RNA using a strand cDNA synthesis kit (Yisheng, China).Then, cDNA was quantified by real-time PCR. GAPDH was used as an internal standard. The primers used are listed in Table 1. All experiments were repeated three times.

**Table 1:** Primer sequences.

Gene	Primer (5ʹ–3ʹ)
Rat Type II collagen A1-F	AAGGGACACCGAGGTTTCACTGG
Rat Type II collagen A1-R	GGGCCTGTTTCTCCTGAGCGT
Rat ADAMTS5-F	CCCAGGATAAAACCAGGCAG
Rat ADAMTS5-R	CGGCCAAGGGTTGTAAATGG
Rat NLRP3-F	CAGACCTCCAAGACCACGACTG
Rat NLRP3-R	CATCCGCAGCCAATGAACAGAG
Rat IL-1β-F	CACCTCTCAAGCAGAGCACAG
Rat IL-1β-R	GGGTTCCATGGTGAAGTCAAC
Rat GAPDH-F	CTCATGACCACAGTCCATGC
Rat GAPDH-R	TTCAGCTCTGGGATGACCTT

### Western blot

The NPCs were washed 3 times with PBS and then lysed with ice-cold RIPA lysis buffer containing PMSF (1:100) (Fudebio, China). After 15 min, each well was scraped with a cell scraper, and the cell lysate was collected into an EP tube, centrifuged at 12,000 rpm, and incubated at 4 °C for 15 min to generate whole-cell extracts. The supernatants were collected. An enhanced BCA protein detection kit (Beyotime, China) was used to determine the protein concentration. A one-step adhesive (Fudebio, China) was used with 10 µl of sample and 10 µl of marker per well (Fudebio, China). Proteins were transferred to a membrane and blocked with 5% skim milk for 2 h before overnight incubation with the following primary antibodies: rabbit anti-collagen II (1:1000; ABclonal), rabbit anti-Sox9 (1:1000; ABclonal), and rabbit anti-GAPDH (1:5000; ABclonal). For removal of excess antibodies, the membrane was washed with TBST three times for 5 min before being incubated with specific horseradish peroxidase-conjugated secondary antibodies (Beyotime, China) for 1 h at room temperature. Three 5-min TBST washes were performed to remove excess secondary antibodies. The intensity of the reaction bands on the membrane was measured using the ChemiDoc Touch Imaging System (Bio-Rad, USA). The experiment was repeated in triplicate.

### RNA-seq

Cells were divided into three groups: control (Con group), 24 h LPS treatment + 1 h ATP treatment (LPS group), and 24 h LPS treatment + 1 h ATP treatment + FG@F (LPS + FG@F group). We extracted RNA from lysed NPCs with TRIzol reagent (Invitrogen, USA). mRNA was purified from 1 μg of total RNA using oligo (dT) magnetic beads, and then, mRNA fragmentation was performed in ABclonal First Strand Synthesis Reaction Buffer. PCR products were purified, and library quality was assessed using an Agilent Bioanalyzer 4150. For sequencing using the NovaSeq 6000 sequencing platform PE150 for read length and the data generated from the Illumina (or BGI) platform for bioinformatics analysis, we chose HISAT2 as the mapping tool for the reference genome. The DESeq2 R package was used to analyse the differential expression between any two groups. The genes with P < 0.05 identified by DESeq2 were considered differentially expressed genes (DEGs). The differentially expressed genes were subjected to enrichment analyses by the clusterProfiler R software packages GO, KEGG and GSEA. The experiments were repeated five times.

### Animal surgery

Shanghai BK Laboratory Animal Co., Ltd. (China), provided SD rats (n = 80, 180–220 g, male). The rat treatment guidelines were approved by the animal ethics committee of Zhejiang Chinese Medical University Laboratory Animal Research Center. Ketamine and xylazine were used to anaesthetize the animals (10:7100 mg/kg i.p.). IVDD was established by puncturing the coccygeal space at C5–6, C6–7, C7–8, and C9–10. After disinfection with iodoalcohol, a 26G needle (diameter = 0.45 mm) was inserted at the level of the annulus fibrosus by palpation and passed through the NP to reach the contralateral annulus fibrosus. After incomplete penetration, the needle was rotated 360° twice for 30 s. All experiments were conducted under sterile conditions. One week after the initial puncture, each group of rats was injected with a 26G needle for treatment. The experiment consisted of 5 groups (n = 8): (1) NC group (no puncture); (2) DC group (puncture and injection of 5 µl of PBS); (3) kaempferol group (puncture and injection of 5 µl of kaempferol); (4) FG group (puncture and injection of 5 µl of FG); (5) FG@F group (puncture and injection of 5 µl of FG@F). Rats were subjected to further assessments at 4 and 8 weeks after treatment. All animal experiments were performed in accordance with the ARRIVE guidelines and regulations.

### Radiological assessment analysis

MicroCT (SkyScan, Belgium) and MRI (Universal Corporation, USA) were performed at 4 weeks and 8 weeks, respectively, to evaluate the signal intensity of the nucleus pulposus and the height of the vertebral space. The T2-weighted signal intensity of the nucleus pulposus of each disc was quantified in MRI images using ImageJ software. MRI images were classified into grades I to IV (I, normal; II, slightly decreased signal intensity but significantly narrowed hyperintense area; III, moderately decreased signal intensity; and IV, severely decreased signal intensity). The disc height of each rat was measured in the CT images using ImageJ (National Institutes of Health, USA).

### Histological and immunohistochemical analyses of IVDD model rats

The rats in each group were overdosed with carbon dioxide at week 4 and week 8. The corresponding disc segment was removed, and the sample was temporarily fixed in 10% neutral buffered formalin. The skin and muscle tissue were removed by dissection, and the sample was then immersed in a decalcified solution (10% EDTA) for 4 weeks. Histological section (8 μm) were prepared using a microtome. H&E staining and Safranin O-fast green (SO) staining were used to assess the extent of IVDD in each group. Two blinded observers assessed the number and morphology of the cells. The grading method was based on a previously reported method^22^. Collagen II was used as an immunohistochemical indicator to assess the degree of disc degeneration. The sample was treated with hydrogen peroxide (3%) for 10 min. The sample was then sealed with 5% BSA at room temperature for half an hour and treated with a mouse anti-rabbit collagen II antibody (1:100; Proteintech) at 4 °C overnight. After 5 washes with PBS, the samples were incubated with biotin-labelled secondary antibodies at 37 °C for 30 min. The staining was developed using the SABC method.

### Statistical analysis

The two groups of data were compared with an independent sample t test, and comparisons of multiple sets of data were performed using one-way ANOVA. All data are expressed as the mean ± standard deviation (SD). P < 0.05 was considered to indicate statistical significance. All intensity signal fluxes and fluorescence expression were calculated using ImageJ software (San Diego, CA, USA). The column graphs were drawn using GraphPad Prism 9 software.

### Ethics approval and consent to participate

The Committee for Zhejiang Chinese Medical University Laboratory Animal Research Center authorised all animal experiments following a robust ethical review.

## Results

### Preparation and characterization of FG@F

**Figure 1:**
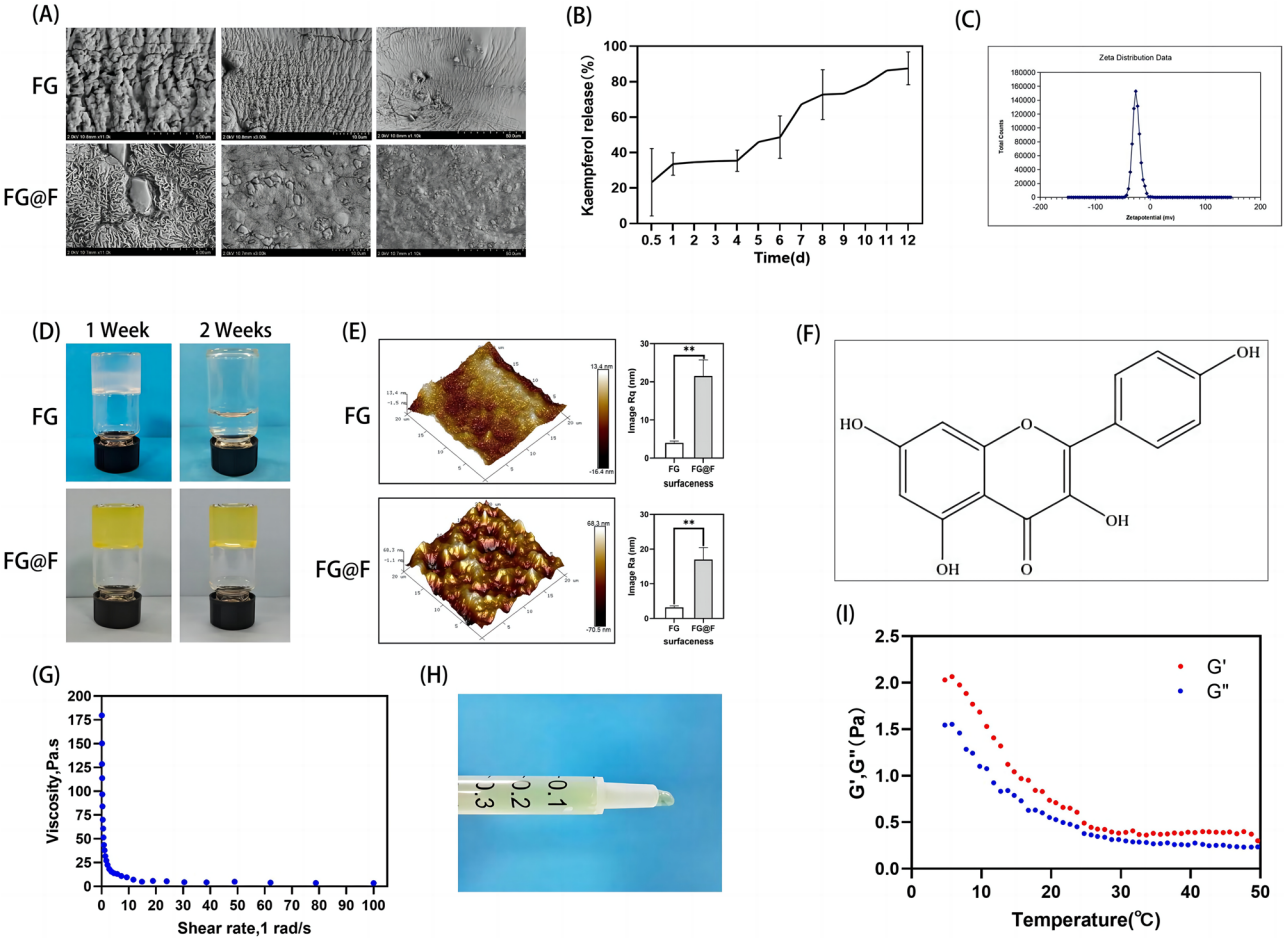
Morphology and characteristics of FG@F. (**A**) SEM analysis of FG and FG@F. (**B**) Control-release curve of FG@F (n = 3). (**C**) Zeta potential of kaempferol (n = 3). (**D**) Images of FG and FG@F. (**E**) Surface roughness of FG and FG@F (n = 3, *P < 0.05). (**F**) Particle size of kaempferol. (**G**) The viscosity changes of FG@F at shear rates of 1 1/s to 100 1/s. (**H**) FG@F injectable image. (**I**) The Gʹ and Gʹʹ changes of FG@F at 4–50 °C.

We succeeded in preparing FG and FG@F. The molecular structure of kaempferol is shown in Fig. 1H. After the samples were freeze dried under a scanning electron microscope (SEM), FG and FG@F were observed (Fig. 1A). Surprisingly, we found that compared with FG, FG@F had greater stability, and after 2 weeks in vitro, FG@F still maintained suitable stability (Fig. 1D). Atomic force microscopy (AFM) showed that compared with FG, FG@F had a rougher surface (Fig. 1E), which verified kaempferol particle adhesion to the FG surface. The molecular structure diagram of kaempferol is shown (Fig. 1F). In addition, the zeta potential of kaempferol was − 25.4 mV (Fig. 1C), and since the surface of FG carries a large number of positively charged groups, kaempferol can strongly bind to it.

### Evaluation of the properties of FG@F

The rheological properties of FG@F were measured to evaluate its mechanical properties. At 37 °C, FG@F showed a Gʹ value higher than Gʹʹ, indicating that it can maintain suitable viscosity in the body (Fig. 1I). As the shear rate gradually increased, the FG@F viscosity continuously decreased, showing that this system had acceptable injection performance (Fig. 1G,H). The slow release curve of FG@F confirmed that it has an excellent ability to slow-release resurrection lily phenol. Within 12 days, FG@F gradually released most of the kaempferol (Fig. 1B).

### Biocompatibility of FG@F in vitro

**Figure 2:**
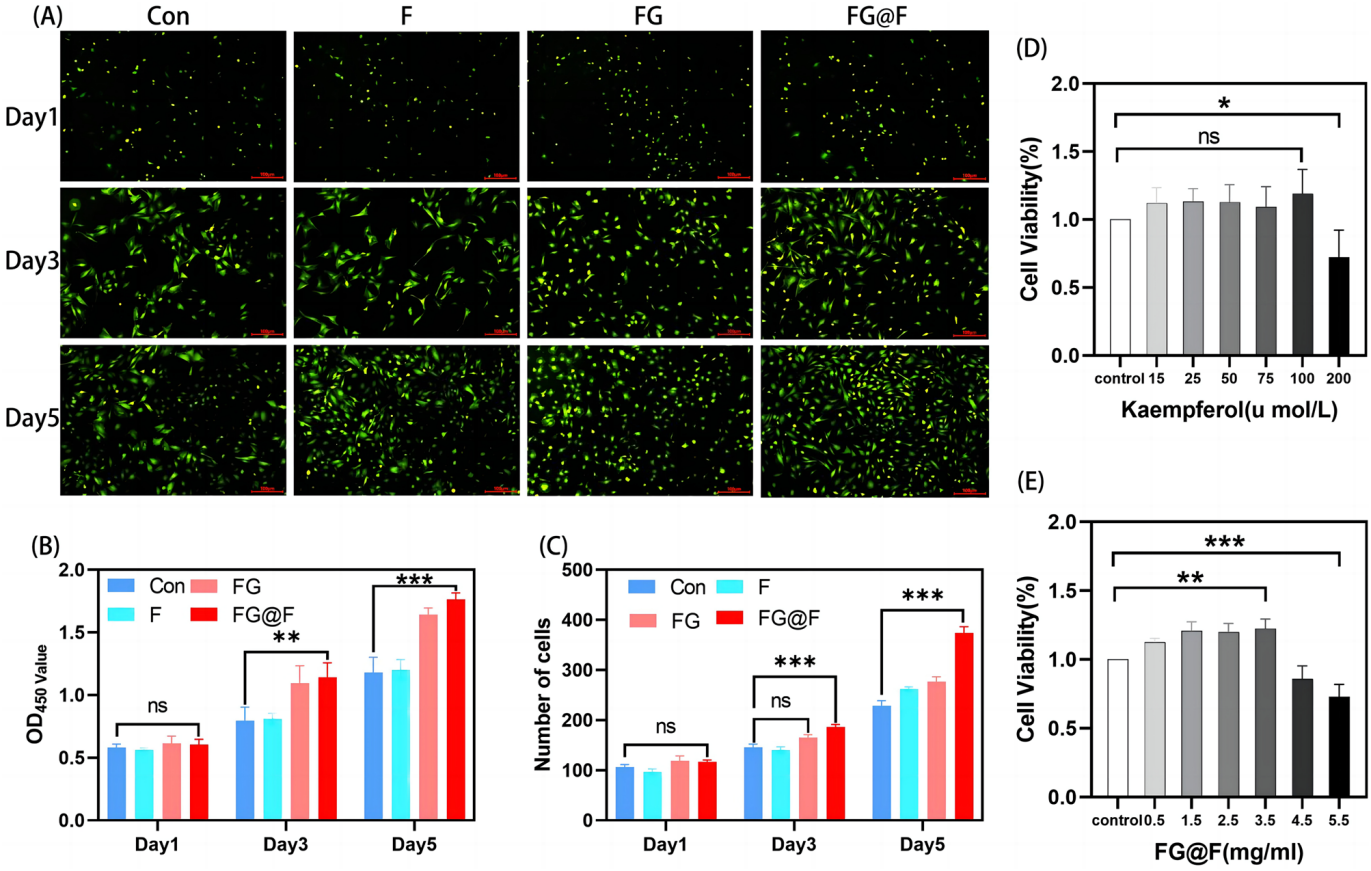
Biocompatibility of FG@F. (**A**) Fluorescence images of living cells (green) in each group. Scale bar = 100 μm. (**B**) The absorbance at 450 nm was compared among the groups (n = 3, *P < 0.05). (**C**) Number of cells cultured with each group (n = 3, *P < 0.05). (**D**) Cell viability of cells cultured with kaempferol (n = 3, *P < 0.05). (**E**) Cell viability of cells cultured with FG@F (n = 3, *P < 0.05).

As shown in Fig. 2D, cell viability was determined by CCK-8 assays, and kaempferol had no obvious cytotoxicity when the concentration was less than 100 µmol/l. Finally, we selected 25 µmol/l as the cell intervention concentration. In the same way, we found that at a fixed concentration of kaempferol, there was no obvious cytotoxicity when the FG@F concentration was less than 3.5 mg/ml, and finally, we chose this concentration as the cellular intervention concentration (Fig. 2E). Immunofluorescence showed that NPC quantity increased with time, and the number of NPCs was significantly higher in the FG@F group than in the NC group (Fig. 2A). The CCK-8 test showed that on the third and fifth days, the cell number was obviously increased in the FG@F group compared with the NC group, and the difference was significant (Fig. 2B). Immunofluorescence showed that there were no significant effects on cell proliferation on Days 1, 3 and 5, indicating that FG@F has good biocompatibility and that FG is an ideal carrier for kaempferol. Over time, the number of NPCs also significantly increased (Fig. 2C).

### The function of FG@F in ECM metabolism

**Figure 3:**
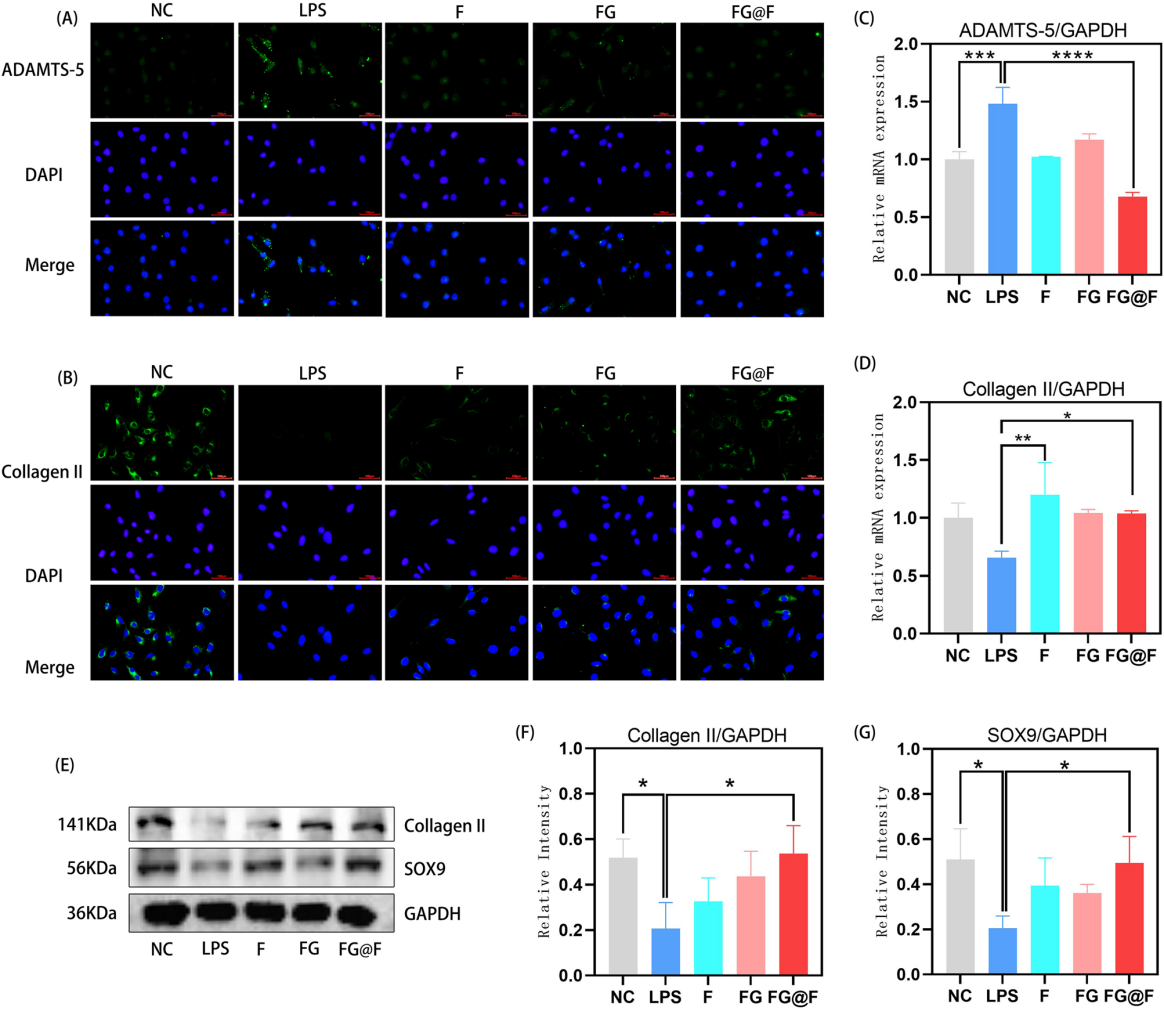
FG@F regulates the matrix metabolism of NPCs. (**A**) Fluorescence images of ADAMTS-5 after LPS + ATP stimulation in the NC, LPS, F, FG and FG@F groups. Scale bar = 100 μm. (**B**) Fluorescence images of collagen II after LPS + ATP stimulation in the NC, LPS, F, FG and FG@F groups. Scale bar = 100 μm. (**C**) RT-qPCR results of ADAMTS-5 after LPS + ATP stimulation among the groups (n = 3, *P < 0.05). Scale bar = 100 μm. (**D**) RT-qPCR results of collagen II after LPS + ATP stimulation among the groups (n = 3, *P < 0.05). (**E**) Protein expression levels of collagen II and Sox9. The original image has been cropped, and full-length blots are presented in Supplementary Figs. 1–3. (**F,G**) Quantification of protein expression. GAPDH served as a loading control (n = 3, *P < 0.05).

To evaluate the effect of FG@F on ECM regulation, we first used RT-qPCR to measure the expression of genes associated with ECM synthesis and degradation in the NPCs of the LPS-treated rats. Compared with the NC group, the LPS group showed decreased expression levels of collagen II in NPCs (Fig. 3D). An important factor promoting intervertebral disc degeneration is the special metalloproteinase gene ADAMTS-5. We observed that the level of ADAMTS-5 in the FG@F group was decreased compared to that in the LPS group (Fig. 3C). The results confirmed that FG@F can affect the metabolism of the ECM to regulate changes in the early disc microenvironment. WB results showed that compared to the LPS treatment, FG@F upregulated the expression of SOX9 and collagen II, affecting the metabolism of ECM (Fig. 3E–G). In addition, the immunofluorescence results showed that FG@F cleaved ADAMTS-5 and increased collagen II levels to promote NPC synthesis in the ECM, leading to ECM accumulation (Fig. 3A,B).

### FG@F inhibits the inflammation of NPCs

**Figure 4:**
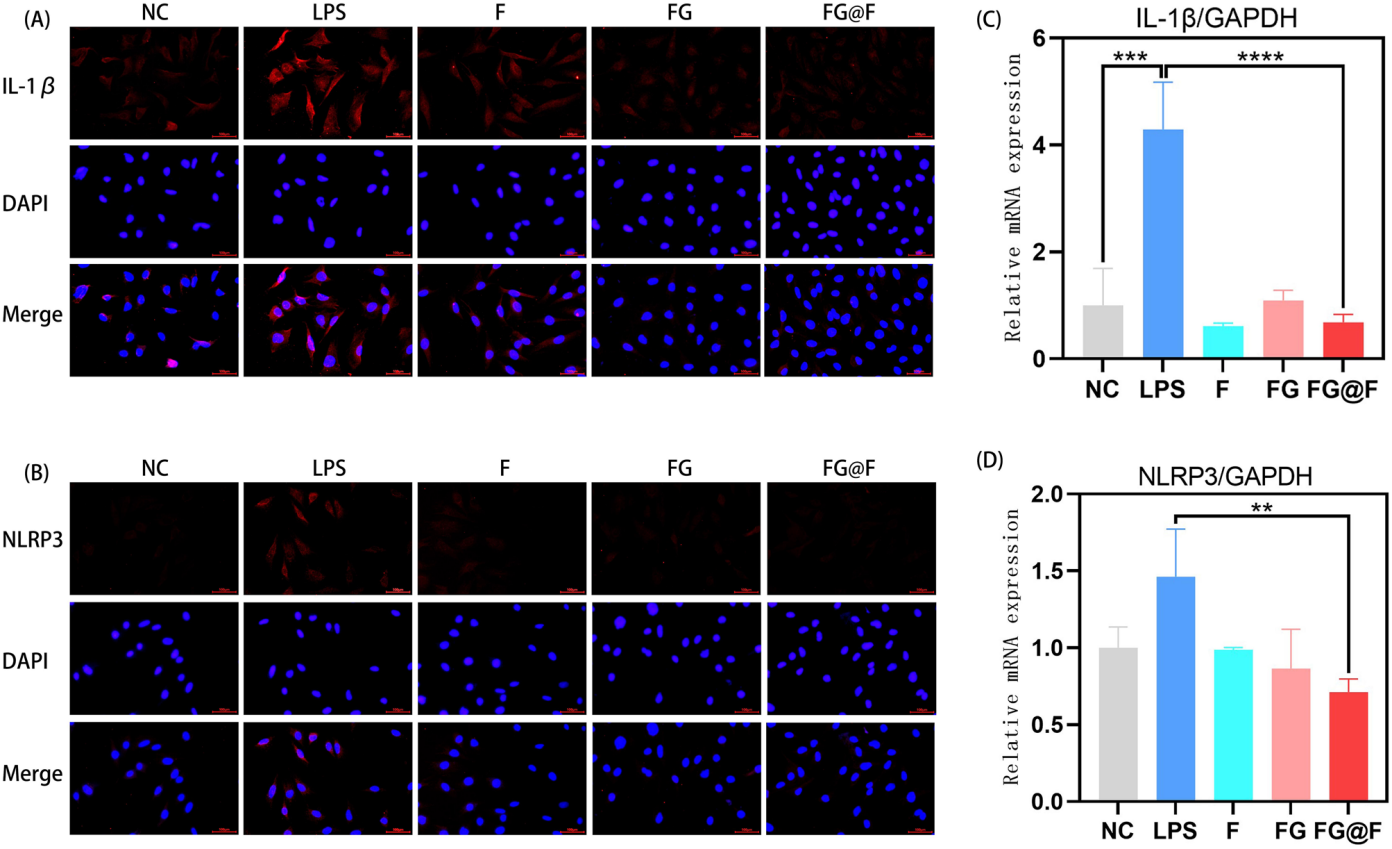
FG@F exerted anti-inflammatory effects on NPCs. (**A**) Fluorescence images of IL-1β expression after LPS + ATP stimulation among the groups. Scale bar = 100 μm. (**B**) Fluorescence images of NLRP3 expression after LPS + ATP stimulation among the groups. Scale bar = 100 μm. (**C**) RT-qPCR results of IL-1β levels after LPS + ATP stimulation among the groups (n = 3, *P < 0.05). (**D**) RT-qPCR results of NLRP3 levels after LPS + ATP stimulation among the groups (n = 3, *P < 0.05).

Using NLRP3 and IL-1β immunofluorescence analysis, we found that the fluorescence intensity after FG@F treatment significantly decreased, and FG@F reduced the inflammation of NPCs (Fig. 4A,B). In addition, we assessed inflammation in NPCs by RT-qPCR. Compared with those in the NC group, the mRNA expression levels of NLRP3 and IL-1β in the LPS group were increased. However, in the FG@F group, the expression levels of NLRP3 and IL-1β decreased (Fig. 4C,D).

### RNA-seq results

**Figure 5:**
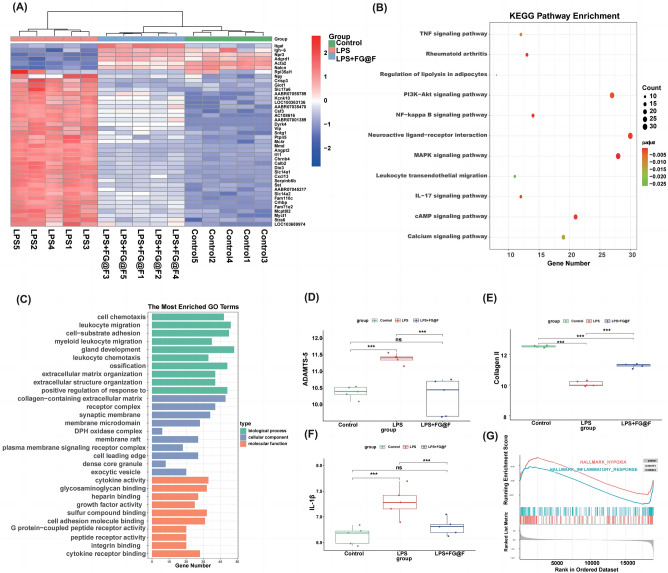
Analysis of transcriptome sequencing results. (**A**) Differential heatmap results among the three groups. (**B**) KEGG enrichment analysis of differentially expressed genes in the LPS group and LPS + FG@F group. (**C**) GO enrichment analysis of differentially expressed genes in the LPS group and LPS + FG@F group. (**D–F**) Differential gene expression among the three groups (n = 5, *P < 0.05). (**G**) Gene set enrichment analysis (GSEA) showed that compared with the control, FG@F exerted a significant inflammatory inhibition effect.

The difference heatmap showed that the LPS + FG@F group and LPS group had significant differences (Fig. 5A). KEGG enrichment analysis of differentially expressed genes between the LPS + FG@F group and the LPS group was performed. The results showed that FG@F can extensively inhibit inflammation-related signalling pathways: the PI3K-Akt signalling pathway, NF-kappa B signalling pathway, MAPK signalling pathway, IL-17 signalling pathway and other signalling pathways (Fig. 5B). GO enrichment analysis of differentially expressed genes was performed in the LPS + FG@F group and LPS group. The results showed biological enrichment in cell chemotaxis, leukocyte migration cell-substrate, and adhesion myeloid leukocyte migration. Cell fractions were enriched in receptor complex synaptic and membrane microdomains. The enriched molecular functions were cytokine activity, glycosaminoglycan binding, heparin binding growth factor activity, sulphur compound binding and adhesion molecule binding (Fig. 5C). The results showed that FG@F could effectively inhibit the expression of IL-1β (Fig. 5F); the addition of FG@F inhibited ADAMTS-5, a gene related to ECM metabolism, and restored the expression of collagen II, a gene related to ECM synthesis (Fig. 5D,E); GSEA showed that FG@F inhibited the inflammatory response (Fig. 5G).

### Imaging evaluation of disc height

**Figure 6:**
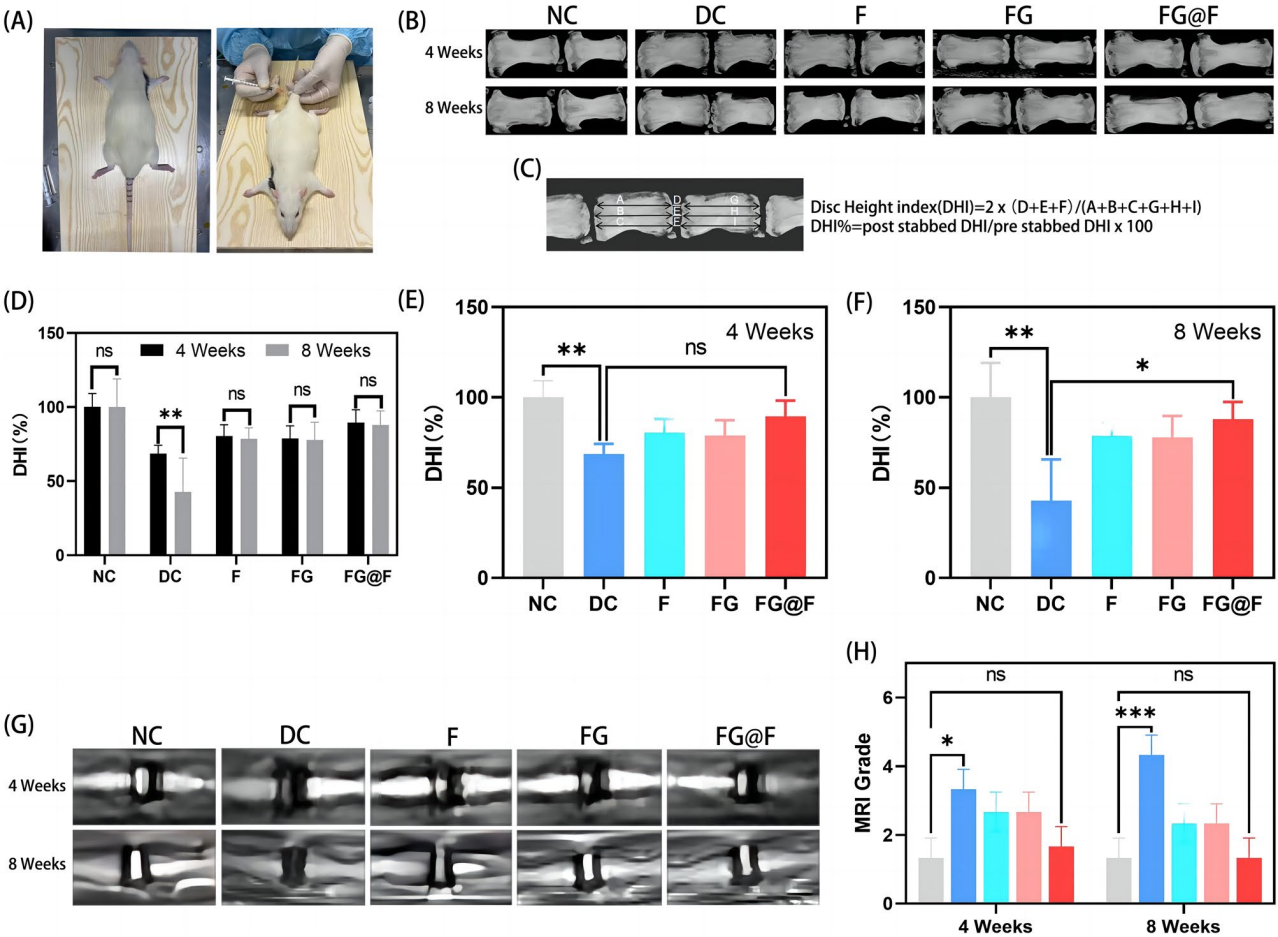
Radiological data of animal experiments. (**A**) Establishment of the caudal vertebrae puncture model in rats. (**B**) Representative X-ray images of the caudal vertebrae of rats at 4 and 8 weeks. (**C**) Calculation method of the disc height index. (**D–F**) The change in DHI% in each group at 4 and 8 weeks (n = 8, *P < 0.05). (**G**) Representative MRI images of the rat caudal vertebrae. (**H**) MRI grading changes in each group at 4 and 8 weeks (n = 8, *P < 0.05).

To explore the repair effect of FG@F on IVDD in rats, we established a model based on needle punctures in the coccyx discs (Fig. 6A). Then, the drug was injected into the intervertebral disc. Each group showed a certain change in disc height over time based on the X-ray analysis (Fig. 6B). The disc height index was measured in CT images using Data Viewer software (Fig. 6C). The disc height index (DHI%) significantly decreased over time in the DC group (Fig. 6D). At different time points, the DHI% of the DC group decreased significantly, and the DHI% of the F and FG groups also decreased to different degrees. The FG@F group, by contrast, showed some disc compression, but compared with the DC, F and FG groups, it still maintained a suitable intervertebral disc height (Fig. 6E,F). As shown by nuclear magnetic resonance (MRI) at 4 and 8 weeks after gavage (Fig. 6G), the NC rats presented a higher T2-weighted signal, indicating increased water content. Significant nucleus pulposus degeneration was observed in the DC group compared with the NC group (P < 0.05), and the intensity of the nucleus pulposus signal in the F, FG and FG@F groups was improved to different degrees compared with that in the DC group (P < 0.05) (Fig. 6H). Therefore, according to the results of the study, the degree of degeneration of the nucleus pulposus was reduced after treatment, which was conducive to the maintenance of vertebral space height.

### Histological and immunohistochemical analyses of the IVDD model rats

**Figure 7:**
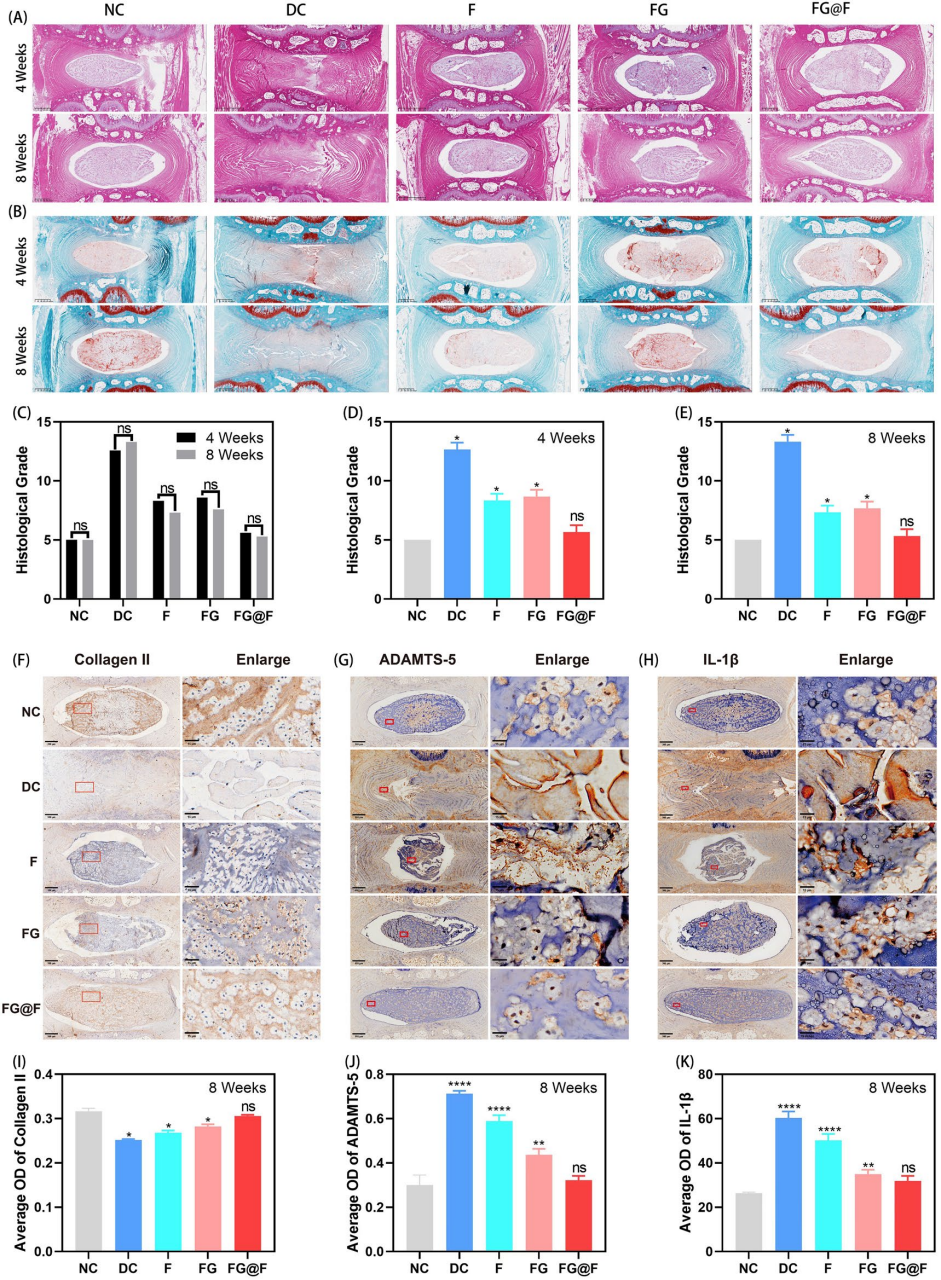
FG@F prevented IVDD. (**A**) H&E staining images of intervertebral disc degeneration in rats at 4 and 8 weeks. (**B**) SO staining at 4 and 8 weeks. (**C–E**) Changes in histological grades at 4 and 8 weeks in each group. (n = 8, *P < 0.05 compared to the NC; *ns* not significant compared to the NC; scale bar = 400 μm). (**F–H**) Immunohistochemical detection of collagen II, ADAMTS-5 and IL-1β at 8 weeks. (**I–K**) The average optical density of collagen II, ADAMTS-5 and IL-1β at 8 weeks. (n = 8, *P < 0.05 compared to the NC; *ns* not significant compared to the NC).

According to the H&E and SO staining results, the NC group maintained complete intervertebral disc morphology, including intact nucleus pulposus tissue and a well-organized AF. Eventually, the DC group showed marked degeneration, resulting in complete fusion. In the F and FG groups, the intervertebral disc degeneration was partially repaired. The FG@F group showed an improved intervertebral structure and form compared with the DC, F and FG groups (Fig. 7A,B). Immunohistochemistry analysis showed that the expression levels of collagen II, ADAMTS-5 and IL-1β in the FG@F group were similar to those in the NC group (P < 0.05), which confirmed the ability of FG@F to improve IVDD (Fig. 7F–K). Histological scoring was performed at weeks 4 and 8 as shown (Fig. 7C–E). The DC group scored the highest, with the most serious degeneration, and the FG@F score was similar to that in the NC group (P < 0.05). This finding shows that FG@F has an optimal effect at 4 weeks, and this effect was maintained for 8 weeks.

## Discussion

In this study, kaempferol-loaded fibrin glue was fabricated to modulate the IVDD microenvironment. The combination of FG and kaempferol in FG@F was more effective than traditional biological scaffolds and is more suitable for the intervertebral disc microenvironment. FG@F can slow the catabolism of ECM by reducing the activity of matrix metalloproteinases. During disc degeneration, slowing the catabolism of ECM corrects the metabolic disorder of ECM and facilitates NPC survival^23–25^. Moreover, the FG@F degradation rate was decreased, and the treatment effect could be prolonged to 12 days. In addition, kaempferol stored in FG@F inhibited the release of inflammatory factors and inactivated the NLRP3 inflammasome. Inhibition of inflammatory cytokines can affect the survival of NPCs.

The disc consists of the nucleus pulposus, annulus fibrosus, and cartilaginous endplate^26^. The function of the intervertebral disc is to provide stability and flexibility to the spine^27,28^. However, IVDD is considered to be an irreversible process in the presence of decreased cell viability, reduced proteoglycan and type II collagen synthesis, and dehydration of the nucleus pulposus^29,30^. FG and kaempferol act together to enhance the tolerance of NPCs to changes in the early inflammatory microenvironment of IVDD and promote their proliferation to a certain extent.

FG@F in this study showed suitable biocompatibility, with treated NPCs showing increased proliferation after 1, 3, and 5 days. Our results show that FG@F can offset the negative impact of substrate metabolic disorder caused by matrix loss and ADAMTS-5 expression. In addition, FG maintained the ECM’s original structure, improved its structural support, and promoted NPC proliferation while resisting inflammatory effects. Most importantly, FG@F can effectively slow the release of kaempferol.

The literature has confirmed that FG contains a large number of positively charged groups^31^. The zeta potential of kaempferol confirmed that it carries a negative charge. By electrostatic attraction, FG can effectively bind kaempferol and promote kaempferol loading.

To further explore the role of FG@F in IVDD, we performed transcriptomic analysis. Our experimental results verified that FG@F showed a good inhibitory effect on inflammation via the NF-kappa B signalling pathway, neuroactive ligand–receptor interaction, MAPK signalling pathway, and TNF-α signalling The inhibition of these pathways and other signalling pathways improved the LPS-induced inflammatory environment. In addition, previous studies have shown that anti-inflammatory therapy is an effective treatment for IVDD because cytokines do not degrade IVD directly, as MMPS or ADAMTS do, but accelerate IVD degeneration by promoting the production of inflammatory substances by disc cells^28^. Our results confirm that FG@F, in addition to its anti-inflammatory effects, can restore ECM synthesis and decomposition balance. Therefore, FG@F may reduce the expression of IL-1β by inhibiting NLRP3, reduce the pyroptosis of NPCs, restore the balance of ECM synthesis and decomposition, and thus alleviate IVDD.

Our results show that FG@F at 5–40 °C can maintain a stable elastic solid form. Premixing kaempferol with fibrin can result in a uniform distribution of the drug, and FG@F after injection of a high modulus allows kaempferol to be fixed in the FG.

FG, as a natural polymer material, is a new treatment material in the field of intervertebral disc degeneration. Previous studies have found that the presence of FG has a positive effect on the expression of collagen II and polymeroglycan, thus contributing to the survival and proliferation of chondrocytes^32–34^. In addition, FG has anti-inflammatory cytokines, which can enhance the anti-inflammatory effect^35,36^. Kaempferol is a widely used flavonoid with optimal anti-inflammatory effects and can promote the proliferation of NPCs^5^. Additionally, its ability to promote the proliferation and growth of NPCs and regulate ECM metabolism was further enhanced by the combination of the two. We found that their combination effectively inhibits disc degeneration, while kaempferol and FG injections alone have limited therapeutic effects.

Compared with that of cell therapy, the immunogenicity of kaempferol and FG is very low, which is the strength of clinical application of FG@F^5,37^. To achieve biocompatibility, we chose biological substances with low rejection rates by the body. Kaempferol is a safe drug approved for the market. Fibrinogen and thrombin are widely available, and if the human body is exposed to them, their harm to the human body is negligible. The FG@F crosslinking process using thrombin is safe. The amount of thrombin is only approximately 1/10 of the prepared solution, and thrombin showed no significant cytotoxicity to NPCs. Our final goal is to develop drug-loaded glues for IVDD treatment.

Nevertheless, this study still has some limitations. We were not able to determine the exact mechanism by which FG@F inhibits NLRP3 aggregation and exerts its anti-inflammatory effects. The specific molecular mechanism by which FG@F delays IVDD deserves further exploration.

## Conclusions

In this study, a new type of kaempferol-loaded fibrin glue was developed with good mechanical strength, sustained release, and low toxicity. Kaempferol loaded into fibrin glue provides a continuous anti-inflammatory effect by inhibiting the aggregation of NLRP3, regulating the ECM metabolism of NPCs, and repairing IVDD. Therefore, FG@F may be a new strategy for IVDD biological therapy.

### Supplementary Information

Supplementary Figures.

## Data Availability

The datasets used and/or analysed during the current study available from the corresponding author on reasonable request.
