# Supplementary material for: Injectable kaempferol-loaded fibrin glue regulates the metabolic balance and inhibits inflammation in intervertebral disc degeneration
Source: Sci Rep. 2023 Nov 15;13:20001. doi: 10.1038/s41598-023-47375-3 (PMC10651831; doi:10.1038/s41598-023-47375-3)
Supplement: Supplementary file 1 — Supplementary Figures. [file 41598_2023_47375_MOESM1_ESM.docx]

**Supplementary Information**

**
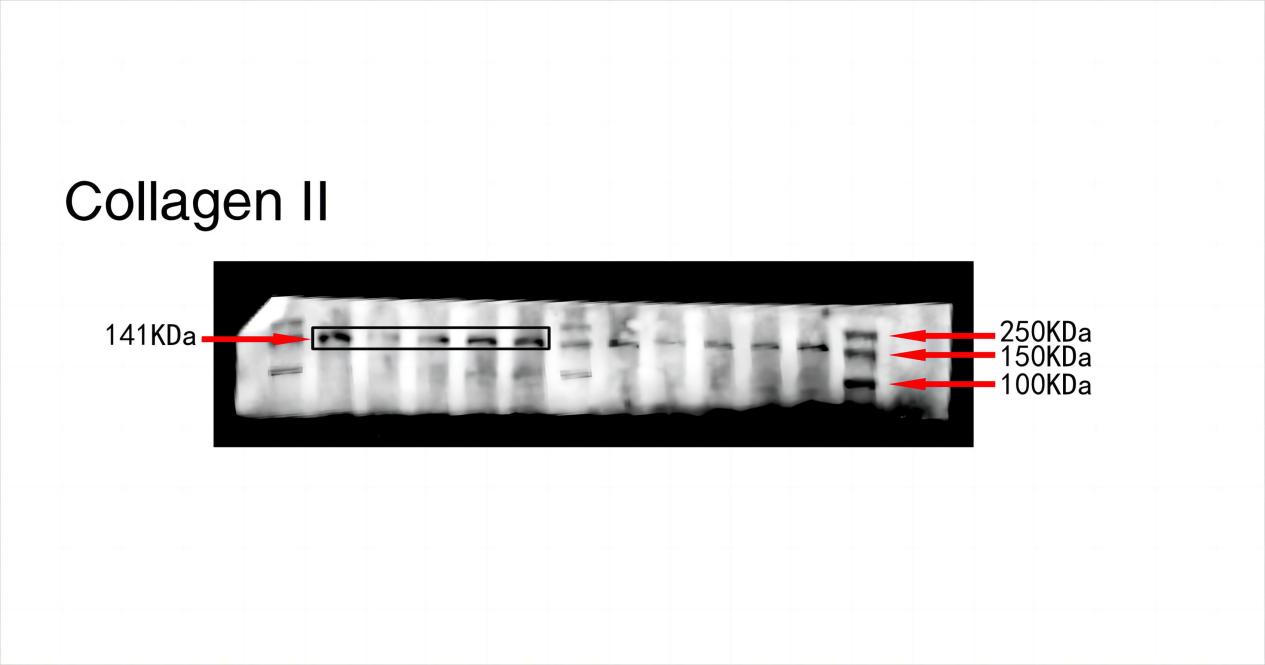
**

**Supplementary Figure 1 . The full-length blots is the display of cropped blots from Figure 3E.**

**
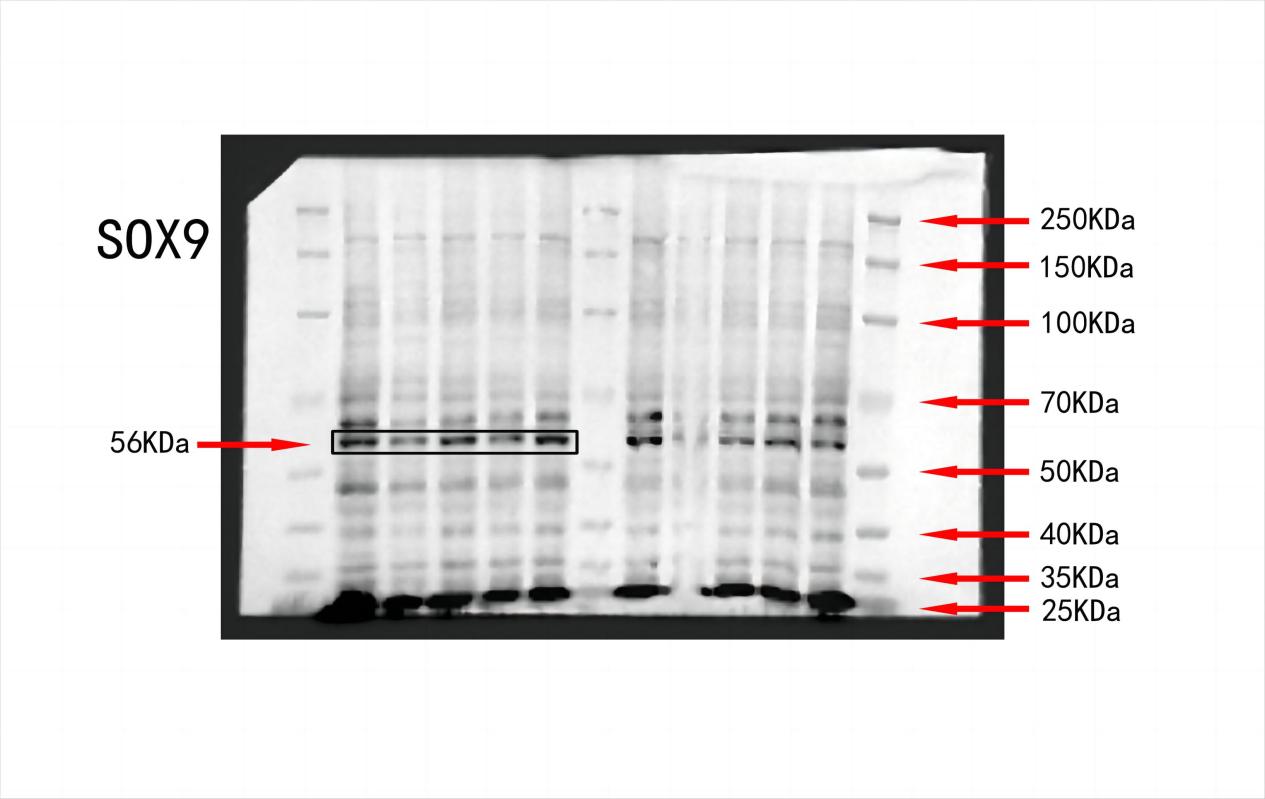
**

**Supplementary Figure 2 . The full-length blots is the display of cropped blots from Figure 3E.**

**
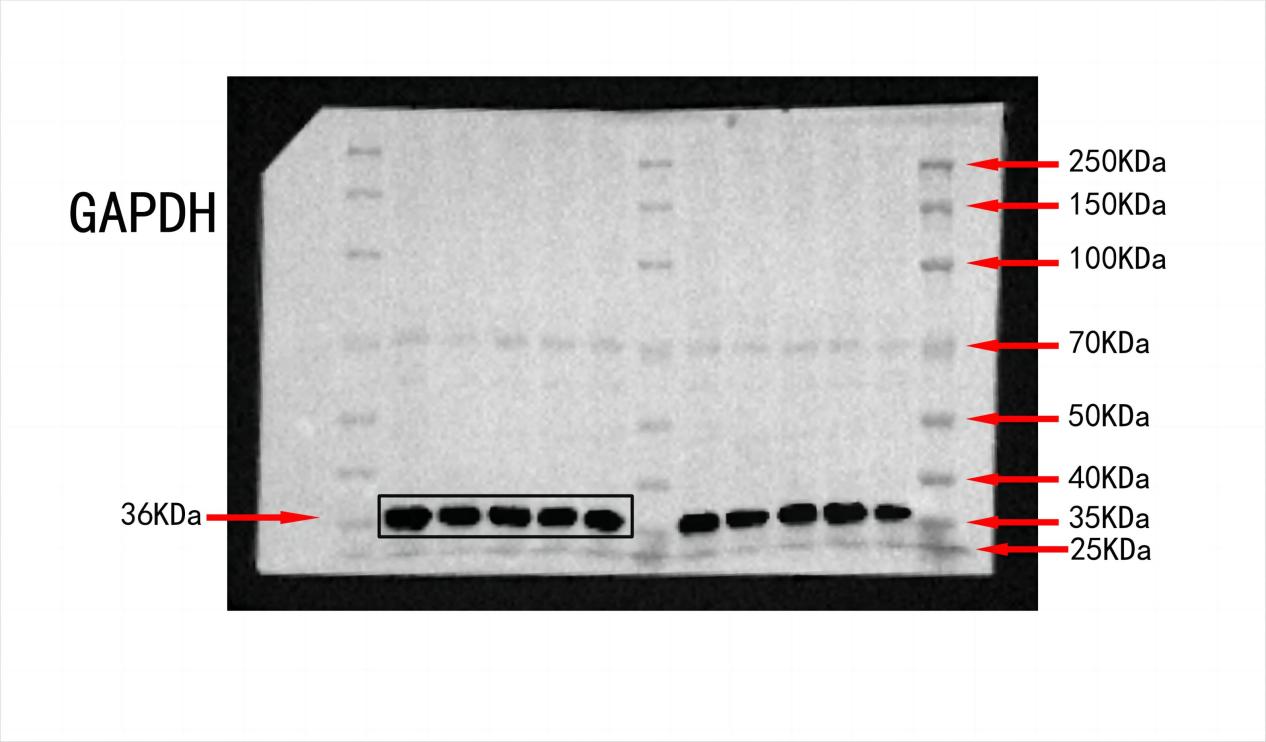
**

**Supplementary Figure 3 . The full-length blots is the display of cropped blots from Figure 3E.**
